# Supplementary material for: The interactome of intact mitochondria by cross-linking mass spectrometry provides evidence for coexisting respiratory supercomplexes
Source: Mol Cell Proteomics. 2017 Dec 8;17(2):216–32. doi: 10.1074/mcp.RA117.000470 (PMC5795388; doi:10.1074/mcp.RA117.000470)
Supplement: Supplemental Data [file supp_RA117.000470_133922_0_supp_23978_fzffwf.zip › spectra_annotation/mito_spectra_annotation/435-1-4-1-6-1.pdf]

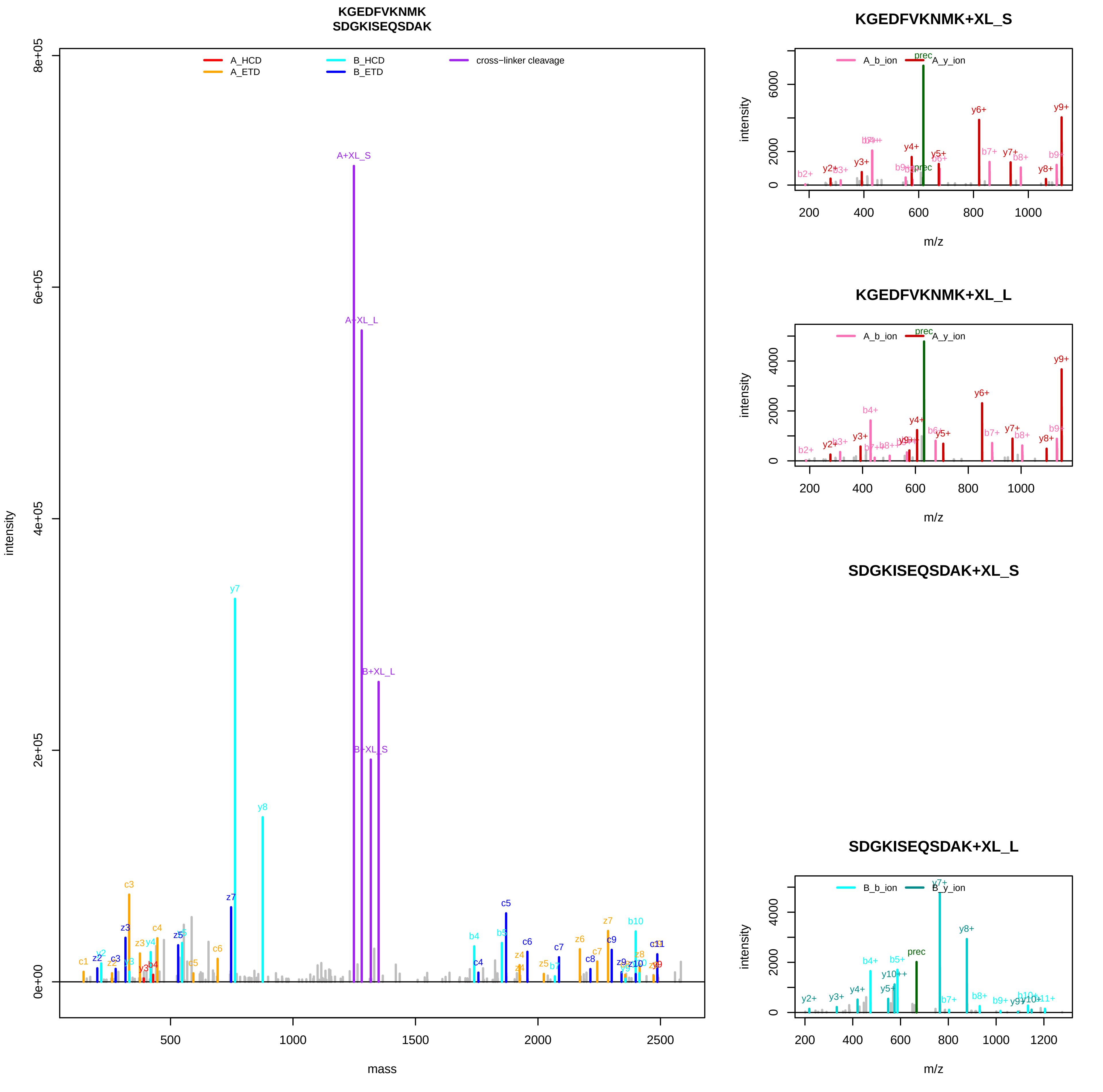

KGEDFVKNMK
SDGKISEQSDAK
A_HCD
A_ETD
B_HCD
B_ETD
cross−linker cleavage
intensity
mass
0e+00
2e+05
4e+05
6e+05
8e+05
500
1000
1500
2000
2500
A+XL_S
A+XL_L
B+XL_L
B+XL_S
y7
y8
c3
z3
z7
c5
b10
KGEDFVKNMK+XL_S
A_b_ion
A_y_ion
prec
y6+
y9+
y4+
y5+
y3+
y2+
b2+
b3+
b6+
b7+
y7+
b8+
y8+
b9+
intensity
m/z
0
2000
6000
200
400
600
800
1000
KGEDFVKNMK+XL_L
A_b_ion
A_y_ion
prec
y9+
y6+
b4+
y4+
y3+
y2+
b2+
b3+
b6+
y5+
b7+
y7+
b8+
y8+
b9+
intensity
m/z
0
2000
4000
200
400
600
800
1000
SDGKISEQSDAK+XL_S
SDGKISEQSDAK+XL_L
B_b_ion
B_y_ion
y7+
y8+
prec
b4+
b5+
y10++
y4+
y5+
y3+
y2+
b7+
b8+
b9+
b10+
y10+
b11+
intensity
m/z
0
2000
4000
200
400
600
800
1000
1200
